# Implementation and use of a national electronic dashboard to guide COVID-19 clinical management in Fiji

**DOI:** 10.5365/wpsar.2023.14.5.967

**Published:** 2023-02-22

**Authors:** Karen Hammad, Sean Casey, Rigamoto Taito, Sara W Demas, Mohita Joshi, Rashmi Rita, Anaseini Maisema

**Affiliations:** a World Health Organization Division of Pacific Technical Support, Suva, Fiji.; b Menzies Health Institute Queensland, Griffith University, Nathan, Queensland, Australia.; c College of Nursing and Health Sciences, Flinders University, Adelaide, South Australia, Australia.; d World Health Organization Regional Office for the Western Pacific, Manila, Philippines.; e School of Population Health, University of New South Wales, Sydney, New South Wales, Australia.; f Lautoka Hospital, Lautoka, Fiji.; g Ministry of Health and Medical Services, Suva, Fiji.; h Office of the Pacific Islands, United Nations Office for the Coordination of Humanitarian Affairs, Suva, Fiji.

## Abstract

**Problem:**

From April to September 2021, Fiji experienced a second wave of coronavirus disease (COVID-19) precipitated by the Delta variant of concern, prompting a need to strengthen existing data management of positive COVID-19 cases.

**Context:**

With COVID-19 cases peaking at 1405 a day and many hospital admissions, the need to develop a better way to visualize data became clear.

**Action:**

The Fiji Ministry of Health and Medical Services, the World Health Organization and the United Nations Office for the Coordination of Humanitarian Affairs collaborated to develop an online clinical dashboard to support better visualization of case management data.

**Outcome:**

The dashboard was used across Fiji at national, divisional and local levels for COVID-19 management. At the national level, it provided real-time reports describing the surge pattern, severity and management of COVID-19 cases across the country during daily incident management team meetings. At the divisional level, it gave the divisional directors access to timely information about hospital and community isolation of cases. At the hospital level, the dashboard allowed managers to monitor trends in isolated cases and use of oxygen resources.

**Discussion:**

The dashboard replaced previous paper-based reporting of statistics with delivery of trends and real-time data. The team that developed the tool were situated in different locations and did not meet physically, demonstrating the ease of implementing this online tool in a resource-constrained setting. The dashboard is easy to use and could be used in other Pacific island countries and areas.

## PROBLEM

From April to September 2021, Fiji experienced its second and largest wave of coronavirus disease (COVID-19), peaking in July 2021 at 1405 cases in one day. The country’s health system was overstretched by COVID-19 testing and triage, with up to 300 hospital admissions per day, reinforcing the need for infection prevention and control measures and resources to treat critical patients. The situation challenged health facilities’ ability to regularly report on hospital census data and management of COVID-19 cases. Lack of timely hospital information made it difficult to monitor adherence to preparedness and response plans and clinical management guidelines developed by the Fiji Ministry of Health and Medical Services (MHMS); it was also difficult to adapt to suit the changing situation at the divisional and national levels. (*1*) It became evident that there was a need to strengthen existing COVID-19 hospitalization reporting systems and data analysis. In addition, visualization of the data in real time could help clinicians and public health staff to respond promptly to the unfolding situation. (*2*)

## CONTEXT

Before the COVID-19 pandemic, the Fiji MHMS used an electronic health information system known as the Patient Information System (PATIS) (*3*) to monitor health service delivery in major hospitals and health centres. However, data from PATIS are summarized manually (*3*) and reported monthly from the subdivision level, which prompted each division (regional area) to develop its own method for COVID-19 hospitalization monitoring and reporting. (*3*-*5*) The various methods were largely paper based and involved increased data entry and analysis so that they could be presented in a PowerPoint format at daily national incident management team (IMT) meetings. The greater workload for health-care workers and the limited capacity for data extraction and analysis meant that a better COVID-19 case management reporting system was needed to enable timely information on COVID-19 admissions from the facility to the national level.

## ACTION

In August 2021, the Fiji MHMS, the World Health Organization (WHO) and the United Nations Office for the Coordination of Humanitarian Affairs (United Nations OCHA) collaborated to create an electronic COVID-19 clinical management dashboard to track COVID-19 case severity, bed occupancy, availability of medical oxygen and oxygen delivery equipment, and management of cases isolating at home. A dashboard is defined as a single-screen visual representation of data from several sources that uses graphics and tables to display qualitative and quantitative indicators. (*6*)

A multidisciplinary team that included clinicians, data experts and epidemiologists from different organizations (including Fiji MHMS, WHO and United Nations OCHA) collaborated remotely to develop the dashboard. Key objectives of the COVID-19 clinical dashboard were to track the isolation and case management of confirmed COVID-19 cases, monitor the application of the clinical care pathway and manage clinical care resources to sustain the country’s existing health-care capacity. An initial prototype of the dashboard was developed using sample data. The prototype was reviewed by the health facilities before further refining the data collection form to facilitate its daily use. To ensure that a technology is usable and achieves its intended purpose, end users must be involved throughout the design process. (*6*) The literature on dashboard conception and design suggests a timeline of 6–12 months; (*7*) however, our dashboard was implemented within 4 weeks.

Data collection was a twofold process: baseline data captured existing health facility capacity to manage COVID-19 cases, then dynamic data captured daily numbers of COVID-19 cases being monitored in hospitals and daily use of resources. The data collection fields captured hospital information, available beds, beds in use, patient occupancy, COVID-19 admissions (disaggregated using WHO clinical severity guidelines), COVID-19 deaths, oxygen availability and oxygen use.

The process of data collection replicated existing processes, with staff appointed by the health facilities or MHMS uploading information about COVID-19 cases isolating in health facilities and in the community to a Google form each day. Access to the Google form link was limited to users verified by the teams at WHO or MHMS, to streamline data entry and prevent errors. The process was piloted in two major hospitals before being expanded to include all health facilities in Fiji. In September 2021, data management officers were recruited at the national level to oversee data quality and assist in monitoring COVID-19 hospital analysis for IMT reporting.

End users were given a link to view the dashboard; this allowed them to view current data from their own device. In this context, end users were nursing and medical heads of hospital departments and public health managers at the Fiji MHMS. As end users became more familiar with the dashboard and the data required to inform clinical and care pathway decision-making, further changes were made to the dashboard. These changes included the addition of home isolation in September 2021, with data on the number of COVID-19 cases isolating at home, their risk for severe disease (high, moderate or low) and recovered cases and deaths. In October 2021, the tool was expanded to track the monitoring and visits to COVID-19 patients in home isolation. Once the consultation period had finished and the dashboard was in consistent use, a nationwide webinar was convened on interpretation of the dashboard and ongoing online support was provided for users.

The dashboard complemented other tools and platforms used during the pandemic response such as daily morning briefs, standard operating procedures and clinical guidelines to inform and support decision-making in the overall response. The dashboard replaced a paper-based system that required time and expertise, and it made visualization of the data easier for the Fiji MHMS. Whereas the paper-based approach to reporting data was punctuated by delays and inconsistencies in reporting, this real-time mode of the dashboard allowed more immediate actions in response to the data.

## OUTCOME

The current iteration of the Fiji dashboard presents information on number of new COVID-19 hospital admissions, positive COVID-19 cases by symptom severity and place of isolation (hospital, non-hospital or home), number of COVID-19-related deaths, use and availability of oxygen resources, and monitoring of the status of positive cases in home isolation. The dashboard is customizable to geographical location, facility type and facility name, enabling all users at local, divisional and national levels to use the same dashboard to meet their needs and inform their response.

### Application at the local level

The dashboard was used by hospitals across the country to guide case management. It provided real-time visibility of COVID-19 patients in hospital and non-hospital isolation. Divisional hospitals could use the dashboard to monitor severe and critical cases at lower-level facilities (e.g. subdivisional hospitals or intermediate care facilities), and identify cases that might require transfer to higher-level care, supporting resource planning.

The dashboard provided further visibility of positive COVID-19 cases in home isolation, which triggered discussions in daily morning briefs about monitoring and management of high-risk patients in home isolation, and assisted in planning home monitoring and referrals. Such discussions helped to identify service gaps such as lack of transport or staff; they also provided the opportunity to assist teams challenged with logistics and other resources.

The dashboard informed the allocation of important resources. For example, disease severity informed the skill mix of hospital staff to match clinical care demands. Oxygen-use data allowed hospital management to source and allocate supplies and necessary equipment to ensure that oxygen was available to patients when needed. Information on disease severity included on the dashboard helped in allocating patients to the most appropriate health facility for the level of care required. Such decisions help facilities and health authorities to make the best use of existing resources.

### Application at the divisional (regional) level

The dashboard allowed clinical and public health managers or leaders to view trends such as increases in COVID-19 cases in health facilities across the country in real time. Thresholds on ward occupancy and oxygen use provided by the dashboard supported decisions to activate surge-capacity plans in anticipation of an increase in demand for resources.

### Application at the national level

At the national level, the dashboard was part of incident management reporting and COVID-19 technical planning meetings. Fiji’s IMT reviewed the dashboard together with COVID-19 surveillance data to monitor and manage the response strategy. Community surveillance data provided information on the scope of the outbreak, while the dashboard highlighted the impact of the outbreak on health-care demand. At the height of the second wave, health facilities quickly reached maximum bed capacity and Fiji’s health-care resources were overstretched. At the national level, this triggered IMT to adapt the national clinical care pathway to prioritize hospitalization of critical and severe COVID-19 cases and introduce home isolation for mild and moderate cases. The dashboard was used to monitor this shift in response strategy, and an overall decline in hospital admissions was seen. As COVID-19 patient admissions declined, facilities could dedicate resources back to non-COVID-19 health-care needs, and the health workforce was better equipped to meet demand.

The dashboard also helped to strengthen communication between the community and health facilities to identify opportunities for improving response mechanisms. Capturing COVID-19 deaths in the dashboard – disaggregated by community, hospital and death before arrival – highlighted where COVID-19 deaths were occurring. An observed rise in deaths before arrival at health facilities led to a mortality review. The review found there were potential delays in seeking care and emphasized the need for increased community engagement and communication on when and how to access care.

**Fig. 1:**
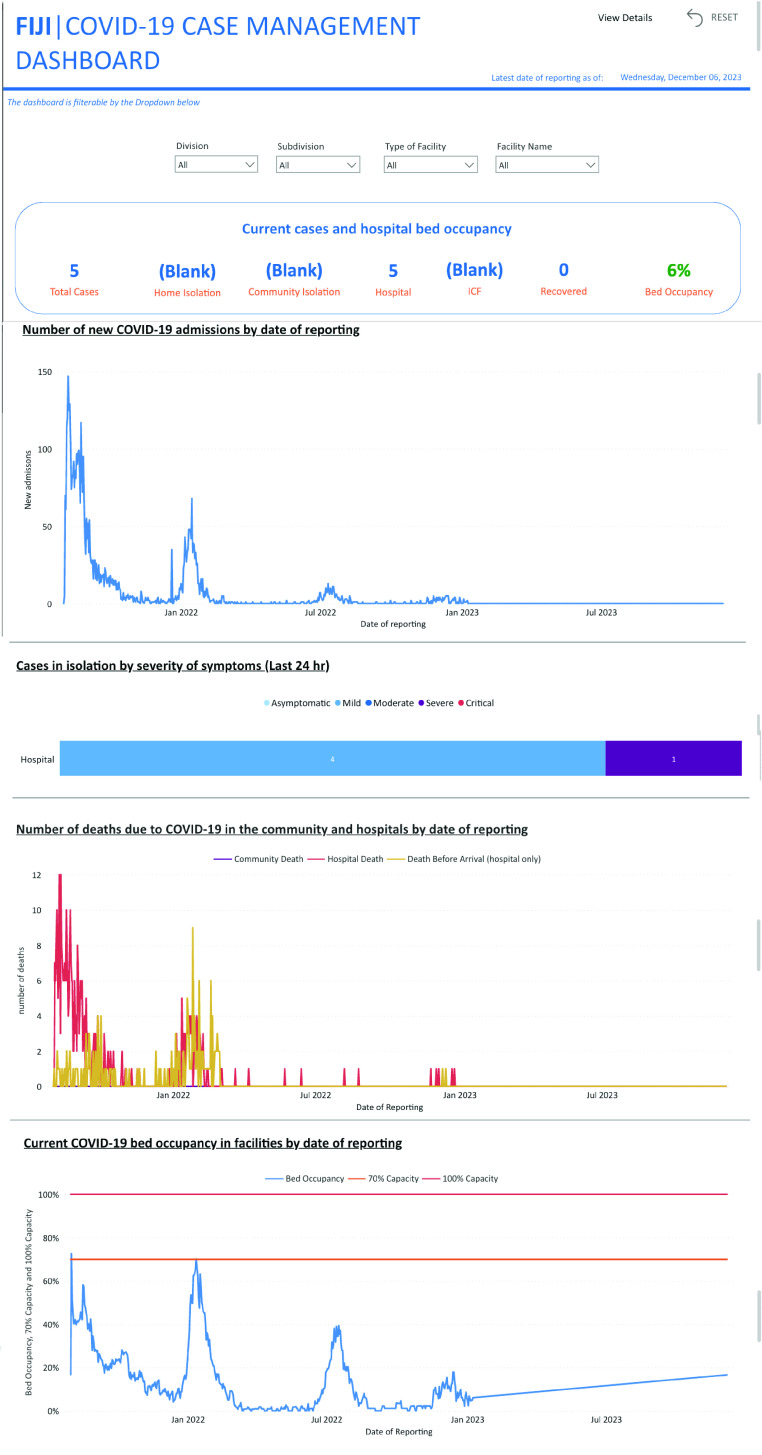
Dashboard interface

**Fig. 2:**
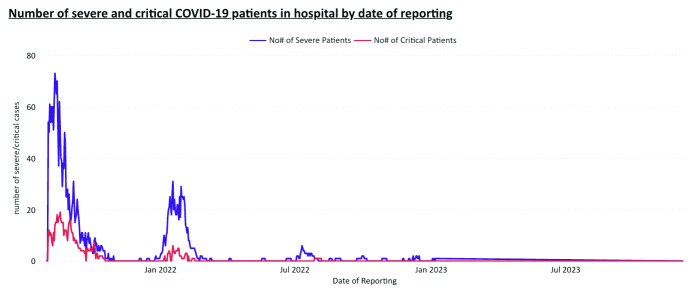
Data capture of hospitalized cases with severe or critical disease severity

The introduction of the dashboard into national COVID-19 reporting and planning provided evidence to guide decision-makers on the necessary interventions to counteract the adverse effects of COVID-19 in Fiji. Examples of data visualization from the electronic dashboard that helped to guide monitoring and clinical management were the number of new cases (**Fig. 1**) and the number of severe and critical cases in hospitals (**Fig. 2**).

[**insert** Figure 1]

[**insert** Figure 2]

### Challenges

Development of the dashboard included some challenges. The development phase involved many hours of discussion between the development team and the health facilities to clarify understanding. There were also challenges related to human resources, with overstretched health facilities expressing difficulty in identifying available staff to collect and enter data. Until processes were established, this led to gaps in data that the development team needed to regularly return to and complete. Another challenge was incorrect interpretation of the dashboard, which occurred when screenshots of the dashboard were used in presentations or the media without context or appropriate interpretation. To address this issue, a short webinar on how to interpret the dashboard correctly was presented to end users, and it was recommended that the dashboard be used only at an operational level.

## Discussion

Since 2020, many dashboards have been created around the world to track and present information on the COVID-19 pandemic; these dashboards have been pivotal in guiding decisions and health system responses. (*6*-*9*) However, much of the literature pertaining to clinical dashboards was published before the pandemic and is fragmented, reporting on different types of dashboards at strategic, tactical and operational levels. (*6*, *10*) One key benefit of an electronic dashboard is that information can be consolidated at a glance to improve decision-making. (*6*, *7*, *10*) Electronic dashboards present a variety of information including patient data such as age, vital signs and oxygen requirements, severity of illness and risk of deterioration (taken from electronic health records); and overall hospital data such as critical care resources, test positivity rate, COVID-19-related bed occupancy and mortality. (*6*, *11*-*14*)

The online dashboard we created allowed key decision-makers to visualize case numbers and place of isolation in real time. Additionally, with many cases isolating at home having risk factors for severe disease, the dashboard provided oversight of this vulnerable group by tracking their level of risk and the date on which the MHMS had last been in contact to check their clinical status.

Communication is critical to an effective and successful pandemic response. Sharing information on the progress of the pandemic helps to inform key stakeholders, for example, by assisting clinical staff with patient care, and helping hospital management and support staff with surge-capacity plans and forecasting logistics, supplies and human resource deployment. Forums such as head of department meetings, executive management meetings and local task force meetings are used to share clinical dashboard trends. Also, Ibrahim et al. (*8*) found that the development and implementation of an electronic dashboard in their health facility enabled physicians to efficiently assess patient volumes and case severity to prioritize clinical care and appropriately allocate services.

There are several important limitations to our dashboard. The first is that we focused on the development and implementation of an electronic dashboard in Fiji. In comparison to other Pacific island countries, Fiji has a relatively large health system that makes it difficult to transfer this online dashboard directly to other country contexts. However, we believe that Fiji’s experience and associated challenges are useful to consider when implementing an electronic dashboard elsewhere. Interpretation of this real-time dashboard also requires a thorough understanding of the dashboard’s data fields, Fiji’s COVID-19 situation and overall response strategy. For instance, an increase in COVID-19 hospitalization seen in June and July 2022 may be due to increased testing, awareness of COVID-19 diagnosis and referral to health facilities. For accurate interpretation, the dashboard should be reviewed in collaboration with other COVID-19 information. Additional limitations included the many hours required to develop the dashboard, incomplete and inconsistent data (particularly following a resurgence of COVID-19), misunderstandings about how the information was collected and efforts to twist the messages that the dashboard presents. (*2*, *15*)

The dashboard is a simple online tool that is easy to use and has applications across different facets of clinical outbreak response. The availability of real-time information via the dashboard facilitates a quick response. Owing to its ease of use, the dashboard can be altered to meet users’ data needs, making this a cost-effective and relatively simple solution for data management and visualization across low-resource settings. It is hoped that the dashboard can be used beyond COVID-19 to track hospital census data and other infectious disease outbreaks.
